# *Mycobacterium caprae* Infection in Captive Borneo Elephant, Japan

**DOI:** 10.3201/eid2410.180018

**Published:** 2018-10

**Authors:** Shiomi Yoshida, Satomi Suga, Satoshi Ishikawa, Yasuhiko Mukai, Kazunari Tsuyuguchi, Yoshikazu Inoue, Taro Yamamoto, Takayuki Wada

**Affiliations:** Nagasaki University Graduate School of Biomedical Sciences, Nagasaki, Japan (S. Yoshida);; National Hospital Organization Kinki-chuo Chest Medical Center Clinical Research Center, Sakai, Japan (S. Yoshida, K. Tsuyuguchi, Y. Inoue);; Fukuyama Zoo, Fukuyama, Japan (S. Suga, S. Ishikawa, Y. Mukai);; Nagasaki University Institute of Tropical Medicine, Nagasaki (T. Yamamoto, T. Wada);; Nagasaki University School of Tropical Medicine and Global Health, Nagasaki (T. Wada)

**Keywords:** Borneo elephant, disseminated tuberculosis, Mycobacterium caprae, Tuberculosis and other mycobacterial infections, regions of difference, whole genome sequence, bacteria, Japan, zoonoses

## Abstract

In 2016, disseminated tuberculosis caused by *Mycobacterium caprae* was diagnosed in a captive Borneo elephant in Japan. The bacterium was initially identified from clinical isolates. An isolate collected during a relapse showed isoniazid monoresistance and a codon 315 *katG* mutation.

Elephants are susceptible to infection by some members of the *Mycobacterium tuberculosis* complex (MTBC) (*1*). The MTBC comprises several genetically homogeneous species that have a wide range of hosts and can cause tuberculosis in humans and in animals. Infection in elephants is presumed to originate from human caretakers who have tuberculosis; however, transmission between elephants or from other animals is also possible (*2*–*4*).

Phylogenetic events during the divergence of MTBC species are represented within the genomes of MTBC species (*5*). Whole-genome sequencing has shown that single-nucleotide polymorphism (SNP) microevolution occurs in MTBC strains in the host.

We describe mycobacterial infection in an elephant that was caused by a relatively uncommon species of MTBC. *M. caprae* infection, a species of the MTBC, was diagnosed in a captive Borneo elephant (*Elephas maximus borneensis*) that was brought directly from Borneo island after being orphaned.

## The Study

In February 2016, an ≈17-year-old female Borneo elephant in the Fukuyama Zoo (Fukuyama, Japan) had low-grade fever (99.9°F), anorexia, progressive weight loss, and cough with sputum. The elephant had been housed alone in a facility with a roofed room and an open-air enclosure and had no contact with other animals. She was seropositive by Chembio DPP VetTB Assay for Elephants (Chembio Diagnostic Systems, Inc., Medford, NY, USA), which detects antibodies to CFP10/ESAT-6 and MPB83 antigens (*6**,**7*). 

Submitted specimens to the National Hospital Organization Kinki-chuo Chest Medical Center (Sakai, Japan) were sputum, feces, urine, and vaginal discharge recovered from the floor. Acid-fast bacilli were visualized on Ziehl−Neelsen staining performed according to standard methods (*8*). Isolates with smooth to greasy, domed, nonchromogenic colonies were recovered from all submitted samples using both 7H11 agar and MGIT broth (Becton Dickinson, Fukushima, Japan) and identified as MTBC using loop-mediated isothermal amplification (Eiken Chemical, Tokyo, Japan) and TaqMan PCR (Roche Diagnostics, Tokyo, Japan) (*9*). 

A single colony was sequenced using MiSeq (Illumina, Inc., Tokyo, Japan). Raw reads were trimmed by base quality and were mapped to the *M. tuberculosis* reference genome, H37Rv (GenBank accession no. CP003248). The initial isolate, EPDC01, was characterized by the presence and absence of genomic regions of difference, the mutation (G to A) of *oxyR*^285^_,_ and *M. caprae*–specific SNPs in *lepA* (*10*) (Table 1). These results unexpectedly suggested that the causative agent of tuberculosis in this elephant was *M. caprae*. The regions of difference analysis suggested that EPDC01 belonged to the Allgäu type of *M. caprae* found in red deer (*Cervus elaphus*) (*11*). However, when we used kSNP3 (*12*), a kmer-based method, to compare SNPs from the entire genome of EPDC01 to previously published Allgäu and Lechtal types of *M. caprae* and other MTBC species, the results showed this isolate was not closely related to either Allgäu or Lechtal types (Figure; Technical Appendix Figure) (*13*).

**Table 1:** Summary of MTBC PCR-typing panel results in a study of *Mycobacterium caprae* infection in a Borneo elephant, Japan, 2016*

Organism	RD9	TbD1	*katG* ^463^	*gyrA* ^95^	RD7	RD8	RD9	RD10	*mmpL6* ^551^	RD12^oryx^	RD1^mic^	RD^can^	RD^seal^	*oxyR* ^285^	RD12^bov^	RD13	RD4	*pncA* ^57^	RD1^BCG^	*lepA*	*katG* ^315^
“Ancentral” *M. tuberculosis*	+	+	CTG	AGC	+	+	+	+	+	+	+	+	+	G	+	+	+	C	+	–	AGC
“Modern” *M. tuberculosis*	+	–	CTG/CGG†	AGC/ACC†	+	+	+	+	+	+	+	+	+	G	+	+	+	C	+	–	AGC
*M. africanum* subtype I (WA-1)	–	+	CTG	AGC	+	+	+	+	+	+	+	+	+	G	+	+	+	C	+	–	AGC
*M. africanum* subtype II (WA-2)	–	+	CTG	AGC	–	+	+	–	+	+	+	+	+	G	+	+	+	C	+	–	AGC
*M. orygis*	–	+	CTG	AGC	–	+	+	–	–	–	+	+	+	G	+	+	+	C	+	–	AGC
*M. microti*	–	+	CTG	AGC	–	+	+	–	–	+	–	+	+	G	+	+	+	C	+	–	AGC
*M. canettii*	–	+	CTG	AGC	–	+	+	–	–	+	+	–	+	G	+	+	+	C	+	–	AGC
*M. pinnipedii*	–	+	CTG	AGC	–	+	+	–	–	+	+	+	–	G	+	+	+	C	+	–	AGC
*M. caprae*	–	+	CTG	AGC	–	+	+	–	–	–	+	+	+	A	–	–	+	C	+	+	AGC
*M. bovis *	–	+	CTG	AGC	–	+	+	–	–	–	+	+	+	A	–	–	–	G	+	–	AGC
*M. bovis* BCG	–	+	CTG	AGC	–	+	+	–	–	–	+	+	+	A	–	–	–	G	–	–	AGC
EPCD01 in this study	–	+	CTG	AGC	–	+	+	–	–	–	+	+	+	A	–	–	+	C	+	+	AGC
EPDC02 in this study	–	+	CTG	AGC	–	+	+	–	–	–	+	+	+	A	–	–	+	C	+	+	ACC

*Includes a differential distribution of known single nucleotide polymorphisms among the MTBC species. MTBC, *Mycobacterium tuberculosis* complex; RD, region of difference; +, positive; –, negative †”Modern” *M, tuberculosis* strains are segregated to 2 and 3 in their respective principal genetic group based on the combinations of *katG*^463^-*gyrA*^95^ polymorphism.

**Figure:**
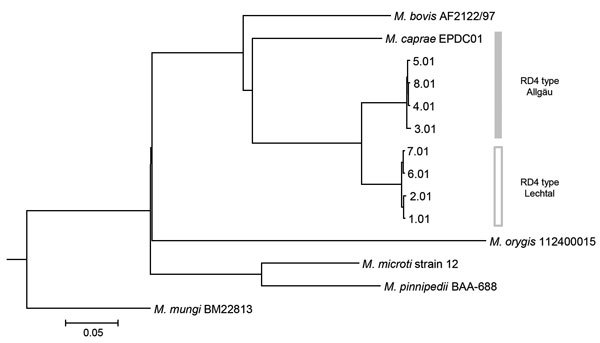
Phylogenetic tree of isolate EPDC01 from a captive Borneo elephant with *Mycobacterium caprae* infection, Japan, 2016, and 8 *Mycobacterium caprae* strains (Allgäu and Lechtal types) from a report by Broeckl et al. (*13*). Short reads of *M. caprae* strains were assembled by CLC Genomics Workbench version 9.5.1 (https://www.qiagenbioinformatics.com/solutions/functional-genomics/?gclid=EAIaIQobChMIvvGL3L7T2wIVTSOBCh2FAAKtEAAYASAAEgKLWvD_BwE) before analysis. Core single-nucleotide polymorphisms of all 13 strains, including reference *M. tuberculosis* complex strains (*M. bovis*, AF2122/97 [GenBank accession no. NC_002945.4]: *M. orygis*, 112400015 [NZ_APKD00000000.1]: *M. pinnipedii*, BAA-688 [MWXB00000000.1]: *M. microti*, strain 12 [CP010333.1]: and *M. mungi*, BM22813 [NZ_LXTB00000000.1]), were determined and used for tree construction based on neighbor-joining by kSNP3 (*12*). A tree including all 61 strains described by Broeckl et al. (*13*) is shown in the Technical Appendix Figure. Scale bar indicates nucleotide substitutions per site.

The isolate was susceptible to isoniazid, rifampin, ethambutol, and levofloxacin according to the broth microdilution method (BrothMIC MTB-1; Kyokuto Pharmaceutical, Inc, Tokyo, Japan). It also was susceptible to pyrazinamide using Bactec MGIT 960 PZA kit (Becton Dickinson).

The infected elephant initially weighed 2,400 kg; isoniazid (4.5–7 mg/kg), pyrazinamide (31–33 mg/kg), and levofloxacin (11 mg/kg) were administered rectally, once a day. When weighing on a scale was not possible, the elephant’s weight was estimated using the chest girth method. After 1 month of treatment, the vaginal discharge disappeared, and the elephant’s sputum culture became negative after 2 months. After 6 months, the multidrug treatment was interrupted for 3 weeks because of severe gastrointestinal disturbance and hepatic dysfunction. Rectal administration of isoniazid and pyrazinamide was resumed for an additional 3 months after recovery from adverse effects. Follow-up trunk wash samples were culture negative, but *M. caprae* was isolated from a sputum sample collected from the chin in February 2017. This new isolate was resistant to isoniazid. The drug regimen was then changed to oral rifampin (10 mg/kg) and rectal ethambutol (30 mg/kg), levofloxacin (10 mg/kg), and pyrazinamide (30 mg/kg). Under the modified treatment, the recurrence symptoms disappeared, and the routine sputum cultures became negative.

We also sequenced the recurrent isoniazid-resistant isolate (EPDC02) using a MiSeq and detected a mutation in *katG* where Ser-315 was replaced by 113 Thr (S315T). No mutations were detected in other representative drug resistance–related genes, such as *rpoB* (rifampin); *rrn*, *gidB*, and *rpsL* (streptomycin); *embABC* (ethambutol); *pncAC* (pyrazinamide); and *gyrAB* (quinolone). The pairwise distance between the initial and relapse isolates (EPDC01 and EPDC02) involved at least 7 single-nucleotide variants found in coding regions (Table 2).

**Table 2:** Single-nucleotide polymorphism differences between isolates EPDC01 and EPDC02 from a captive Borneo elephant with *Mycobacterium caprae* infection, Japan, 2016*

Genomic position	Gene	Mutation	Amino acid change	Description of function
1,338,135	*Rv1194c*	G → frameshift	Gln127 → frameshift	Unknown
1,445,272	*Rv1290A*	C → T	Ala72 → Val	Unknown
2,155,168	*katG*	C → T	Ser315 → Asn	Catalase; isoniazid resistance
2,577,556	*Rv2306A*	G → A	Gly150 → Glu	Unknown
3,200,585	*Rv2891*	T → G	Leu107 → Arg	Unknown
3,777,062	*Rv3365c*	C → A	Gly147 → Val	Unknown
4,298,329	*pks2*	G → A	Pro426 → Leu	Lipid metabolism
4,345,372	*Rv3869*	G → C	Ala112 → Pro	Unknown

*Excluded paralogue genes, which are the descendants of an ancestral gene and underwent a duplication event.

For serologic monitoring of treatment, we tested archived serum samples collected before and during treatment using the DPP assay. Although a sample in November 2003 was reactive on CFP10/ESAT-6 but not on MPB83, at the time of culture-based tuberculosis diagnosis (February 2016), the reaction to CFP10/ESAT-6 was more intense, and the complete band of MPB83 appeared. The intensity to 2 test lines gradually decreased during the initial treatment (September 2016) and disease recrudescence (February 2017). The modified 18-month course of treatment is scheduled to end in October 2018.

Employees at the zoo were assessed for tuberculosis based on symptoms, radiographs, and serology using the QuantiFERON-TB Gold test (QIAGEN, Tokyo, Japan); none had active tuberculosis. Clinical examination, culture, PCR, and tuberculin skin test were used to evaluate as many primates and hoofstock as possible in the collection, and no tuberculosis-positive animals were identified.

## Conclusions

A better understanding of tuberculosis in elephants is crucial to improve medical management and reduce risk of transmission to other animals and humans. During the initial treatment of the elephant reported here, treatment was interrupted for 3 weeks because of adverse effects. Reducing the dose of isoniazid was the probable cause of the acquired isoniazid resistance. When treating tuberculosis in elephants, the benefits and adverse effects should be weighed carefully. The DPP provides an indirect measure of infection and disease status (*6*). In this elephant, declining DPP reactivity was thought to indicate a response to therapy; however, more sensitive biomarkers to monitor therapeutic response are needed.

Previously, epidemiologic observations of elephant tuberculosis by IS*6110* restriction fragment-length polymorphism have been based on evidence of local zoonotic risk for transmission to humans or of an epizootic reservoir for transmission to elephants or other animal species (goats and rhinoceros) (*1**,**14*). Recently, *M. tuberculosis* strains in 2 captive elephants in a small traveling circus harbored 3 nucleotide changes, according to whole-genome sequencing (*15*). *M. tuberculosis* has been isolated from Asian elephants among regions in southern Asia (*3**,**4*). Although the transmission routes have not been defined, our result and those of previous reports indicate that MTBC species may be spilling over into elephants.

Our finding emphasizes the need to identify the species of MTBC when tuberculosis is diagnosed in elephants. Although corroborating epidemiologic evidence of transmission has not been discovered, genomic data of the *M. caprae* isolates has been registered in the open database (BioProject ID PRJDB6469; BioSample ID SAMD00098240 for EPDC01, SAMD00098241 for EPDC02). Accumulation of genomic data of clinical isolates is expected to be helpful for future comparative studies.

Technical Appendix Phylogenetic tree of isolate EPDC01 and 61 *Mycobacterium caprae* strains.
