# Supplementary material for: Mycobacterium caprae Infection in Captive Borneo Elephant, Japan
Source: Emerg Infect Dis. 2018 Oct;24(10):1937–40. doi: 10.3201/eid2410.180018 (PMC6154153; doi:10.3201/eid2410.180018)
Supplement: Technical Appendix — Phylogenetic tree of isolate EPDC01 and 61 Mycobacterium caprae strains. [file 18-0018-Techapp-s1.pdf]

Article DOI: https://doi.org/10.3201/eid2410.180018

Mycobacterium caprae Infection in Captive Borneo Elephant, Japan

Technical Appendix

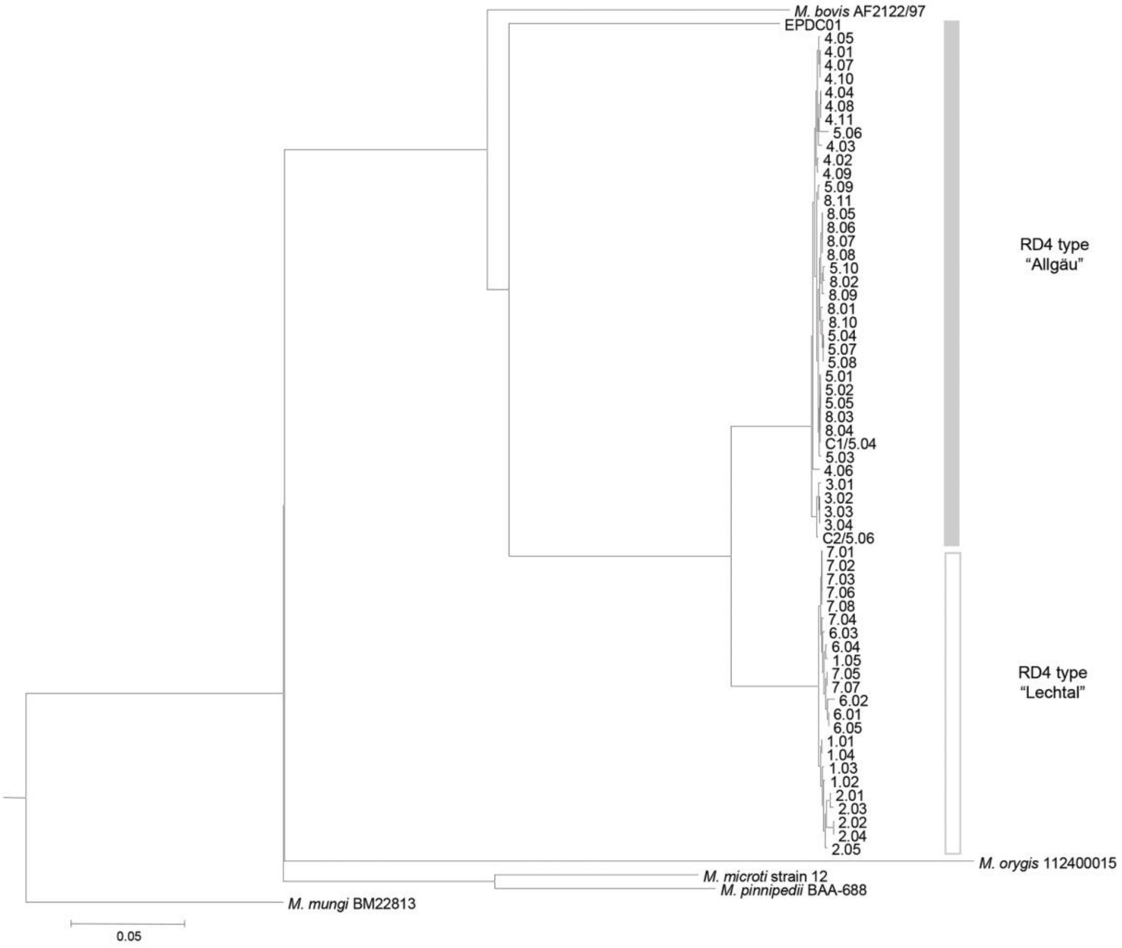

Technical Appendix Figure. Phylogenetic tree of isolate EPDC01 from a captive Borneo elephant with *Mycobacterium caprae* infection, Japan, 2016, and 61 *Mycobacterium caprae* strains (Allgäu and Lechtal types) in Broeckl et al. (*1*). Short reads of *M. caprae* strains were assembled by CLC Genomics Workbench, version 9.5.1 (https://www.qiagenbioinformatics.com/solutions/functional-

genomics/?gclid=EAIaIQobChMIvvGL3L7T2wIVTSOBCh2FAAKtEAAYASAAEgKLWvD_BwE) before analysis. Core single nucleotide polymorphisms of all 13 strains, including reference *M. tuberculosis* complex strains (*M. bovis*: AF2122/97 [GenBank accession no. NC_002945.4], *M. orygis*: 112400015 [NZ_APKD00000000.1], *M. pinnipedii*: BAA-688 [MWXB00000000.1], *M. microti*: strain 12 [CP010333.1], and *M. mungi*: BM22813 [NZ_LXTB00000000.1]) were determined and used for tree construction based on neighbor-joining by kSNP3 (*2*). Scale bar = nucleotide substitutions per site.

References

1. Broeckl S, Krebs S, Varadharajan A, Straubinger RK, Blum H, Buettner M. Investigation of intra-herd spread of *Mycobacterium caprae* in cattle by generation and use of a whole-genome sequence. Vet Res Commun. 2017;41:113–28. PubMed http://dx.doi.org/10.1007/s11259-017-9679-8

2. Gardner SN, Slezak T, Hall BG. kSNP3.0: SNP detection and phylogenetic analysis of genomes without genome alignment or reference genome. Bioinformatics. 2015;31:2877–8. PubMed http://dx.doi.org/10.1093/bioinformatics/btv271
